# The Inflammasomes

**DOI:** 10.1371/journal.ppat.1000510

**Published:** 2009-12-24

**Authors:** Mohamed Lamkanfi, Vishva M. Dixit

**Affiliations:** 1 Department of Physiological Chemistry, Genentech, South San Francisco, California, United States of America; 2 VIB Department of Medical Protein Research, and Department of Biochemistry, Ghent University, Ghent, Belgium; University of California San Francisco, United States of America

## Caspase-1 Cleaves Interleukin (IL)-1β and IL-18 Once Activated in Inflammasomes

Innate immune cells such as macrophages and dendritic cells produce potent inflammatory cytokines to mount an appropriate immune response against microbial threats. The related cytokines interleukin (IL)-1β and IL-18 are generated as cytosolic precursors that require cleavage by the cysteine protease caspase-1 to generate biologically active IL-1β and IL-18. Hence, mice lacking caspase-1 are defective in the maturation and secretion of IL-1β and IL-18 [1]. Caspase-1 itself is generated as an inactive precursor protein that contains a “caspase activation and recruitment domain” (CARD) motif in its N-terminus, which is essential for bringing two or more zymogens sufficiently close to induce their autocatalytic activation, a process believed to occur in large cytosolic protein complexes termed “inflammasomes”.

Most inflammasomes contain a member of the nucleotide binding and oligomerization domain (NOD)-like receptor (NLR) family. These proteins are thought to function as sensors that detect conserved microbial components in intracellular compartments, similar to the role of mammalian Toll-like receptors (TLRs) at the cell surface and within endosomes [2]. NLRs share a domain organization that usually includes (1) an amino-terminal protein–protein interaction domain such as a CARD or pyrin domain; (2) an intermediary NACHT domain that is required for nucleotide binding and self-oligomerization; and (3) a variable number of carboxy-terminal leucine-rich repeat (LRR) motifs involved in sensing pathogen molecules. In general, the pathogen-associated molecular patterns (PAMPs) recognized by NLRs and TLRs are vital for microbial survival, representing either nucleic acid structures unique to microbes or cell wall components alien to mammalian cells.

The bipartite adaptor protein ASC plays a central role in the interaction between NLRs and caspase-1 in each of these inflammasome complexes. As a consequence, caspase-1 activation and the production of IL-1β and IL-18 are abolished in ASC-deficient macrophages that are infected with intracellular bacteria or stimulated with a combination of microbial ligands and ATP [3]. ASC has a specific role in caspase-1 activation because secretion of the cytokines TNF-α and IL-6 is not affected by ASC deficiency.

**Figure 1:**
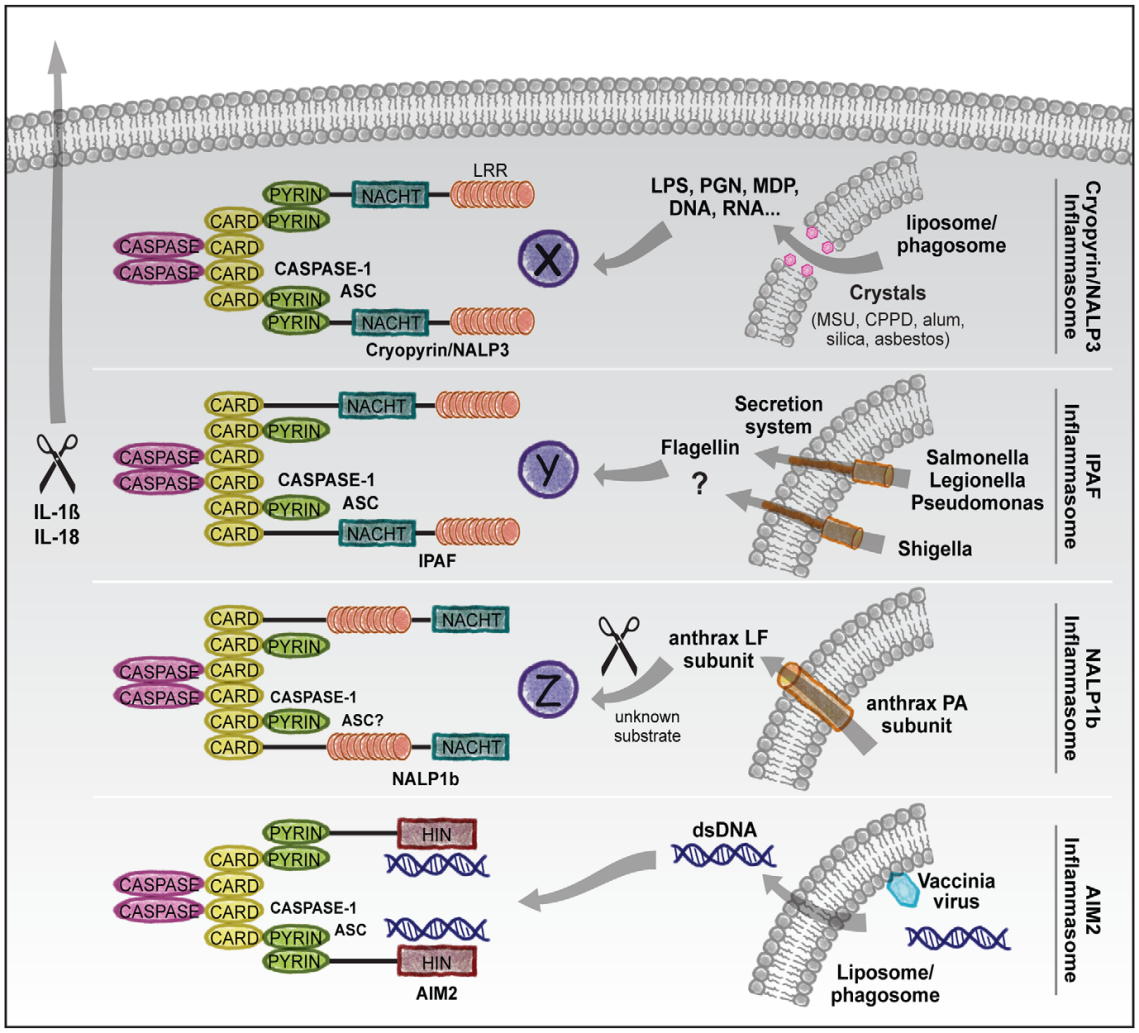
Stimuli and composition of distinct inflammasomes. The NLR proteins NALP1b, Cryopyrin/NALP3, and IPAF and the HIN-200 protein AIM2 assemble a caspase-1 activating inflammasome complex in response to specific microbial or bacterial factors. The murine NALP1b inflammasome recognizes the cytosolic presence of anthrax LT. The Cryopyrin/NALP3 inflammasome recognizes multiple PAMPs in combination with ATP or nigericin, as well as crystalline substances including MSU, silica, and asbestos particles. The IPAF inflammasome senses *Salmonella* and *Legionella* flagellin and a yet unidentified *Shigella flexneri* compound, which all access the cytosol through a type III or IV secretion system. Cytosolic PAMPs may trigger assembly of a particular inflammasome complex by causing modifications in unknown host factors (X, Y, Z) that are monitored by specific NLR proteins. In contrast, AIM2 directly binds dsDNA in the cytosol to induce caspase-1 activation. The CARD/pyrin-containing adaptor protein ASC is essential for all these inflammasome complexes, although its role in the NALP1b inflammasome remains to be formally established. Once activated, caspase-1 processes the IL-1β and IL-18 precursors into the mature cytokines, which are secreted through an unknown mechanism.

Genetic studies in mice suggest that at least four inflammasomes of distinct composition are formed in vivo in a stimulus-dependent manner (Figure 1): the IPAF inflammasome [3]–[5], the NALP1 inflammasome [6], the Cryopyrin/NALP3 inflammasome [7]–[9], and a fourth inflammasome triggered by *Francisella tularensis* infection [8],[10]. Biochemical studies suggested the existence of an additional inflammasome containing NALP2 [11],[12], although specific ligands for this inflammasome remain to be identified. In addition to these NLRs, the HIN-200 protein absent in melanoma 2 (AIM2) was recently shown to trigger caspase-1 activation in response to cytoplasmic double-stranded DNA (dsDNA) [13]–[16].

## The IPAF Inflammasome

Caspase-1 activation is largely abolished in IPAF-deficient macrophages infected with *Salmonella typhimurium* [3]–[5], *Legionella pneumophila* [17],[18], *Pseudomonas aeruginosa* [19]–[21], or *Shigella flexneri* [22]. Bacterial flagellin, which typically is translocated into the cytosol by a bacterial secretion system (type III for *S. typhimurium* and *P. aeruginosa*; type IV for *L. pneumophila*), was identified as the bacterial compound that is sensed by IPAF. *S. flexneri* lacks flagellin, however, so the nature of the *S. flexneri* factor that is sensed by IPAF is unknown. Nevertheless, the finding that recombinant purified flagellin induces IPAF-dependent caspase-1 activation when delivered to the cytosol, either using pore-forming proteins or upon transfection with cationic lipids, indicates that cytosolic flagellin is sufficient for IPAF activation regardless of its delivery mechanism [4],[5],[23]. Interestingly, the extracellular flagellin receptor TLR5 is not required for IPAF-mediated detection of cytosolic flagellin and the subsequent activation of caspase-1 [4],[5], suggesting that TLR5 and IPAF have evolved to control distinct signalling pathways (NF-κB activation and caspase-1 activation, respectively) when the host is infected with intracellular pathogens.

## The NALP1 Inflammasome

The *Bacillus anthracis* lethal toxin (LT) consists of a pore-forming protective antigen (PA) subunit and a metalloprotease subunit lethal factor (LF). PA allows delivery of LF into the cytosol of infected cells [24]. Macrophages from C57BL/6J and multiple other inbred mice strains are resistant to LT-induced death, whereas 129/S1 macrophages are highly susceptible. Mutations in the *Nalp1b* gene were identified as the key determinant of LT susceptibility in mice [6]. Five distinct *Nalp1b* alleles were identified in 18 mouse strains analyzed, demonstrating the extremely polymorphic nature of the *Nalp1b* gene [6]. Two alleles were found in the susceptible mouse strains, whereas the remaining three alleles correlated with LT resistance. In addition, morpholinos targeting NALP1b rendered LT-sensitive macrophages resistant to killing. Importantly, LT-induced toxicity was restored in C57BL/6 macrophages by expressing the susceptible *Nalp1b* allele from a 129/S1-derived bacterial artificial chromosome (BAC) [6]. This study established that a functional *Nalp1b* allele is required for LT to induce cell death in mouse macrophages. Interestingly, caspase-1 is activated in LT-sensitive but not in LT-resistant macrophages. Moreover, caspase-1-deficient macrophages are protected from LT-induced death, even in the presence of a sensitive *Nalp1b* allele [6]. Recently, the proteolytic activity of the anthrax LF subunit was shown to be required for NALP1b-mediated caspase-1 activation [25], but the identity of the LF substrate(s) that are processed in order for caspase-1 to be activated remains obscure.

## The Cryopyrin/NALP3 Inflammasome

Gain-of-function mutations within the NACHT domain of the NLR protein Cryopyrin/NALP3 are associated with three autoinflammatory disorders characterized by skin rashes and prolonged episodes of fever in the absence of any apparent infection. These hereditary periodic fever syndromes are Muckle-Wells syndrome (MWS), familial cold autoinflammatory syndrome (FACS), and neonatal-onset multisystem inflammatory disease (NOMID), and they are collectively referred to as the Cryopyrin/NALP3-associated periodic syndromes (CAPS) [12]. Functional studies revealed that the disease-associated Cryopyrin/NALP3 mutations enhance caspase-1 activation and IL-1β secretion [26]. Indeed, mononuclear cells from CAPS patients spontaneously secrete IL-1β and IL-18 [12], and IL-1 receptor antagonists have proved to be an effective treatment for these autoinflammatory syndromes [27]. In addition to the CAPS-associated mutations in Cryopyrin/NALP3, polymorphisms in regulatory elements that cause decreased Cryopyrin/NALP3 expression and IL-1β production were recently linked with increased susceptibility to Crohn's disease in humans [28].

Human monocytes constitutively express active caspase-1 and only require stimulation with TLR ligands such as LPS, peptidoglycan, or microbial RNA for secretion of mature IL-1β [29]. In contrast, co-exposure to millimolar concentrations of ATP is required for activation of the Cryopyrin/NALP3 inflammasome in primary human and mouse macrophages, dendritic cells, and the leukemic cell line THP-1 [7]–[9],[29]. ATP triggers opening of the non-selective cation channel of the purinergic P2X_7_ receptor, and this is followed by the gradual opening of a larger pore attributed to the hemichannel pannexin-1, which is recruited upon P2X_7_ receptor activation [30]–[33]. Knockdown and pharmacological inhibition of pannexin-1 indicates that the hemichannel protein is critical for Cryopyrin/NALP3-dependent caspase-1 activation and IL-1β secretion in response to LPS+ATP [32]. Cryopyrin/NALP3 also mediates caspase-1 activation in macrophages infected with *Staphylococcus aureus* or adenovirus [8],[34]. In addition, medically relevant crystals such as monosodium urate (MSU), calcium pyrophosphate dihydrate (CPPD), crystalline asbestos, and silica were shown to induce Cryopyrin/NALP3-dependent activation of caspase-1. The Cryopyrin/NALP3 inflammasome was hence suggested to participate in the aetiology of gout, pseudogout, asbestosis, and silicosis [35]–[38]. Moreover, the Cryopyrin/NALP3 inflammasome was proposed to be required for antibody production with alum-containing vaccines [39]–[41], but this has been disputed by others [42],[43]. Because of this plethora of molecularly diverse agonists, activation of the Cryopyrin/NALP3 inflammasome is widely believed to involve the generation/activation of a common secondary messenger. Although the precise nature of this factor remains elusive, several mechanisms have been suggested, including K^+^ efflux [44],[45], lysosomal destabilization [37], and the generation of reactive oxygen species [36],[38].

## The AIM2 Inflammasome

Transfection of dsDNA was recently shown to induce caspase-1 activation through the HIN-200 family member AIM2 [13]–[16]. The HIN domain in AIM2's C-terminus directly interacts with dsDNA, whereas the N-terminal pyrin domain recruits caspase-1 through ASC. Interestingly, the dsDNA vaccinia virus relies on the AIM2 inflammasome for caspase-1 processing [16], whereas DNA-dependent activation of caspase-1 by adenoviral particles required Cryopyrin/NALP3 [34]. Studies with additional dsDNA viruses may reveal the intricacies of these inflammasomes.

## The *Francisella*-Sensing Inflammasome

The Gram-negative coccobacillus *Francisella tularensis* is the causative agent of tularaemia. Infected mice lacking ASC or caspase-1 show markedly increased bacterial burden and mortality when compared to their wild-type counterparts, indicating that caspase-1 activation plays a vital role in the normal immune response to this pathogen [10]. *F. tularensis* mutants that cannot escape the vacuole are incapable of activating caspase-1, thus linking phagosomal escape to caspase-1 activation [46]. Type I interferon signalling functions upstream of *F. tularensis*–induced caspase-1 activation [47], which further requires ASC, but neither Cryopyrin/NALP3 nor IPAF [8],[10]. This suggests the existence of a separate *F. tularensis*–sensing inflammasome.

## Concluding Remarks

It is evident that inflammasomes fulfill important roles in the innate immune response. An open question that currently drives inflammasome research is how inflammasomes are activated. One possibility is a direct ligand–receptor interaction, as recently shown for activation of the AIM2 inflammasome by cytosolic dsDNA [13]–[16]. Activation of other inflammasomes may also be direct or rather rely on the generation of a secondary messenger that is recognized by a specific inflammasome (Figure 1). However, the molecular nature of such cellular “danger signals” remains an enigma. Elucidating how inflammasomes are activated will provide new insights into the mechanisms governing immunity and may pave the way for new therapeutic approaches for autoimmune disorders.
